# Molecular Heterogeneity of Pediatric AML with Atypical Promyelocytes Accumulation in Children—A Single Center Experience

**DOI:** 10.3390/genes14030675

**Published:** 2023-03-08

**Authors:** Aleksandra Borkovskaia, Sofia Bogacheva, Tatiana Konyukhova, Elina Dadakhanova, Marina Gaskova, Olga Soldatkina, Maria Dubrovina, Alexander Popov, Ekaterina Mikhailova, Evgenia Inushkina, Marat Kazanov, Evgeniy Matveev, Galina Novichkova, Michael Maschan, Alexey Maschan, Yulia Olshanskaya, Elena Zerkalenkova

**Affiliations:** 1 Dmitry Rogachev National Medical Research Center of Pediatric Hematology, Oncology and Immunology, Samora Maschela Str. 1, 117998 Moscow, Russia; 2 Moscow Regional Oncology Hospital, Karbisheva Str. 6, 143900 Balashikha, Russia; 3 Institute for Information Transmission Problems (the Kharkevich Institute, RAS), Bolshoy Karetny per. 19, bld. 1, 127051 Moscow, Russia; 4 Skolkovo Institute of Science and Technology, Bolshoy Boulevard 30, bld. 1, 121205 Moscow, Russia

**Keywords:** AML, atypical promyelocytes, fusion genes, *RAR* gene family, *KMT2A*

## Abstract

Acute promyelocytic leukemia (APL) pathogenesis is based on *RARA* gene translocations, which are of high importance in the diagnosis of and proper therapy selection for APL. However, in some cases acute myeloid leukemia (AML) demonstrates APL-like morphological features such as atypical promyelocytes accumulation. This type of AML is characterized by the involvement of other *RAR* family members or completely different genes. In the present study, we used conventional karyotyping, FISH and high-throughput sequencing in a group of 271 de novo AML with atypical promyelocytes accumulation. Of those, 255 cases were shown to carry a typical chromosomal translocation t(15;17)(q24;q21) with *PML::RARA* chimeric gene formation (94.1%). Other *RARA*-positive cases exhibited cryptic *PML::RARA* fusion without t(15;17)(q24;q21) (1.8%, *n* = 5) and variant t(5;17)(q35;q21) translocation with *NPM1::RARA* chimeric gene formation (1.5%, *n* = 4). However, 7 *RARA*-negative AMLs with atypical promyelocytes accumulation were also discovered. These cases exhibited *TBL1XR1::RARB* and *KMT2A::SEPT6* fusions as well as mutations, e.g., *NPM1* insertion and non-recurrent chromosomal aberrations. Our findings demonstrate the genetic diversity of AML with APL-like morphological features, which is of high importance for successful therapy implementation.

## 1. Introduction

Acute promyelocytic leukemia (APL) is a subvariant of acute myeloid leukemia (AML). Distinctive features of APL are the accumulation of atypical promyelocytes and chromosomal translocations affecting the *RAR* family genes [1]. The *RAR* genes encode the retinoic acid steroid receptors of three isotypes: RARA, RARB and RARG, which differ in their ability to bind to the SMRT corepressor [2]. The RARA (retinoic acid receptor α) isotype is a transcriptional repressor that predominates in cells and binds to the RXR (retinoid X receptor). Normally, this heterodimeric complex with attached corepressors (SMRT, N-CoR, Sin3a) and HDAC histone deacetylase binds to a specific DNA region—*RARE* (retinoid acid responsive element)—thus blocking RNA synthesis in the absence of retinoic acid. Upon activation of the RARA receptor by retinoic acid, its conformation changes, eventually leading to normal myeloid cell differentiation [3,4].

More than 90% of APL carry a chromosomal translocation t(15;17)(q24;q21) with *PML::RARA* chimeric gene formation, where *PML* is a tumor suppressor gene on chromosome 15 [3,5]. Such a translocation is typical for both the classical hypergranular AML-M3 and the hypogranular M3v, in accordance with the FAB classification [6,7,8]. PML-RARA inhibits the activity of genes responsible for hematopoietic differentiation, which leads to impaired maturation of promyelocytes, their accumulation in the bone marrow and impaired apoptosis. Three types of *PML::RARA* were identified with different breakpoints in *PML*: bcr1 in intron 6, bcr2 in exon 6 and bcr3 in intron 3 (58–75%, 5–10% and 15–33%, respectively), while *RARA* gene rupture occurs in intron 2 [9,10,11]. *PML::RARA* may also form as a result of cryptic chromosomal material exchange between chromosomes 15 and 17 with no t(15;17)(q24;q21) detectable by karyotyping or FISH [12].

In 1–2% of APL, *RARA* may fuse to other partners forming t(11;17)(q23;q21)/*ZBTB16::RARA*, t(5;17)(q35;q21)/*NPM1::RARA*, t(11;17)(q13;q21)/*NuMa::RARA*, t(17;17)(q11;q21)/*STAT5b::RARA*, etc. [9,13,14]. However, in some cases of AML with cytological characteristics closely resembling M3 or M3v FAB morphology, no *RARA* rearrangement can be detected. This group of *RARA*-negative AML with APL-like morphological features is either associated with other *RAR* family genes rearrangements such as *RARB* and *RARG* [2] or lacks any *RAR* rearrangements at all [5,15]. The diagnosis of these AML types is difficult, and therapy with all-trans retinoic acid (ATRA) and arsenic trioxide (ATO) is not effective due to the lack of specific drug binding sites [1,16]. Therefore, variant *RARA* rearrangements as well as *RARB* and *RARG* cases exhibit an unfavorable prognosis. In the current work, we aimed to study tumor-specific genetic lesions in AML with APL-like morphological features, with a focus on *RARA*-negative cases.

## 2. Materials and Methods

### 2.1. Study Cohort

From 2010 to 2022, 271 cases of de novo AML with atypical promyelocytes were investigated at the Dmitry Rogachev National Research Center of Pediatric Hematology, Oncology and Immunology (age 0 to 18 y.o., median 10 y.o., male:female ratio 1:1, typical hypergranular M3 *n* = 126, hypogranular M3v *n* = 47, M3 not specified *n* = 98). Further characterization was carried out using immunophenotypic, cytogenetic and molecular genetic methods.

### 2.2. Morphological Examination

Samples were selected by the morphology of bone marrow (BM) smears corresponding to the M3 or M3v FAB subtype [6,7,8]. All obtained BM smears were stained according to Pappenheim–Kryukov for a general assessment of cell morphology. According to the FAB classification [6], there are two morphological variants of APL: hypergranular (M3) 60–70% of APL and hypogranular (M3v). However, in either form, atypical promyelocytes with irregularly shaped nuclei or abnormal dicots are seen.

The hypergranular variant is characterized by an excess of cytoplasmic azurophilic granules and numerous Auer rods (faggot cells). The hypogranular type is represented by a low content or absence of granules in the cytoplasm of promyelocytes, as well as single faggot cells containing 1–3 Auer rods [13]. If there was an equal percentage of cells of both forms or if due to a small number of promyelocytes in smear cases they could not be attributed to specific variant, they were recorded in the group “M3 not specified”.

In addition, to accurately differentiate AML from APL and monocytic leukemia, all samples were subjected to cytochemical analysis consisting of 5 reactions: assessment of myeloperoxidase (MPO) activity, Sudan black (SB) staining for lipids, Schiff (PAS) reaction, assessment of nonspecific esterase and suppression of esterase with sodium fluoride. Promyelocytes have strong staining in reactions to MPO and SB, in contrast to undifferentiated blasts. In addition, in APL, the PAS reaction demonstrates the diffuse distribution of the dye, and the excess of nonspecific esterase in myeloid cells is not suppressed by sodium fluoride.

### 2.3. Flow Cytometry

Diagnostic immunophenotyping was performed as described previously [17]. The antigen expression profile of tumor blasts was investigated by 6–10-color MFC using three-laser flow cytometers: FACS Canto II (Becton Dickinson, BD, San Jose, CA, USA) or Navios (Beckman Coulter, BC, Indianapolis, IN, USA). EuroFlow guidelines for machine performance monitoring were used [18]. Cytometer Setup and Tracking Beads (BD) and Flow-Check Pro Fluorospheres (BC) were used for daily cytometer optimization. The fluorochrome-conjugated monoclonal antibodies used were listed previously [19]. At least 50,000 nucleated cells were acquired. Flow cytometric data were analyzed with Kaluza 2.1 software (BC). Residual normal lymphocytes were used as control cells for the positivity/negativity definition. Leukemic cell gating was performed mainly according to dim CD45 expression and appropriate side-scatter (SSC) values [17]. Positivity thresholds were set as 20% for surface antigens and 10% for intracellular antigens [17].

### 2.4. Cytogenetics and Molecular Genetics

BM aspirate obtained at diagnosis was cultured overnight without mitogenic stimulation and processed as previously described [20]. G-banded karyotyping was performed in accordance with An International System for Human Cytogenomic Nomenclature (2020) [21].

FISH screening for t(15;17)(q24;q21) was performed with Kreatech PML-RARa DCDF probe (Leica Microsystems B.V., Amsterdam, The Netherlands) in any newly diagnosed AML. Cases with atypic promyelocytes by morphological examination but *PML::RARA*-negative by FISH were further analyzed with Kreatech *RARA* DCBA probe (Leica Microsystems B.V., Amsterdam, The Netherlands) for other types of *RARA* rearrangements. Kreatech *KMT2A* DCDF probe (Leica) was used for all patients with additional chromosomal abnormalities and patients without metaphase plates. All FISH procedures were performed according to the manufacturer’s instructions and at least 100 interphase nuclei were analyzed.

Total DNA and RNA were simultaneously extracted from the BM samples using the InnuPrep DNA/RNA Mini Kit (Analytik Jena AG, Jena, Germany). Reverse transcription-polymerase chain reaction (RT-PCR) with the Biomed multiplex system for *PML::RARA* was used in routine [22]. *NPM1::RARA* was analyzed with a nested PCR system [23,24]. The same qPCR and nested PCR systems were used for follow-up monitoring. The primers used are listed in Table S1.

Samples negative for *RARA* gene rearrangements by either method were subjected to whole transcriptome sequencing with NEBNext Ultra II Directional RNA library preparation kit (NEB, Ipswich, MA, USA) and sequenced on Illumina NextSeq (Illumina, San Diego, CA, USA). Alignment to NCBI preformatted GRCh38 reference genome [25] was performed using STAR (ver. 2.7.9a) [26]. Fusion transcripts were detected using Arriba (ver. 2.1) [27]. Additional somatic mutations were detected with Human Myeloid Neoplasms NGS panel (Qiagen, Hilden, Germany). Single-nucleotide variants were called with Pisces (ver. 5.3) [28] and annotated with InterVar (ver. 2.0.2) [29]. Short indels were called with Pindel [30]. A *KMT2A*-positive sample was also subjected to LDI-PCR [31]. Fusion-specific primers for direct genomic DNA PCR and RT-PCR were used for follow-up monitoring. The primers used are listed in Table S1.

### 2.5. Statistics

Overall survival (OS) was defined as the duration from the date of diagnosis to death or last follow-up. Event-free survival (EFS) was defined as the time from diagnosis to the first event—relapse or death from any cause. Survival rates were estimated with the Kaplan–Meier method [32], and standard errors were calculated according to the Greenwood method. Differences in outcome between groups were compared using the log-rank test. ExcelStat-16 was used to perform statistical analysis.

## 3. Results

According to morphological examination, most patients had hypergranular M3 AML (46.5%, 126 of 271). Hypogranular M3v AML was found in 17.3% (47 of 271), and in 36.2%, M3 and M3v could not be differentiated (98 of 271).

Standard karyotyping was successful in 69.4% cases (188 of 271). T(15;17)(q24;q21) was found to be a sole abnormality in 64.4% (121 of 188), while it conjoined with additional abnormalities in 23.4% (44 of 188), and had complex structure in 3.7% (7 of 188; Table S2). In 5.9% no t(15;17)(q24;q21) was found (11 of 188).

The presence of *PML::RARA* was confirmed by FISH in 94.1% patients (255 of 271). Of those, 173 were studied by routine qRT-PCR. It revealed bcr3 to be the most common (50.3%, 87 of 173), followed by bcr1 (37.6%, 65 of 173) and bcr2 (6.4%, 11 of 173). In 5.8% of patients, the type of *PML::RARA* was not determined (10 of 173).

No translocation between chromosomes 15 and 17 was found in 5.9% (16 of 271). Additional studies with RARA DCBA were performed and 1.48% *RARA*-rearranged *PML::RARA*-negative cases were uncovered (4 of 271). All of them carried NPM1::RARA transcript detected by nested RT-PCR.

Patients *RARA*-negative by either FISH approach (4.43%, 12 of 271) were first analyzed by routine qRT-PCR. This uncovered 1.9% cases of cryptic 15q25 and 17q21 chromosomal material exchange leading to *PML::RARA* formation (5 of 271) transcribed as bcr3 and bcr1 subtypes (*n* = 3 and 2, respectively). This left seven cases negative for any type of detectable *RARA* rearrangement. They were further subjected to RNAseq which revealed only 0.8% of unconventional fusion transcripts (TBL1XR1::RARB and KMT2A::SEPT6, 1 of 271 each).

**Figure 1:**
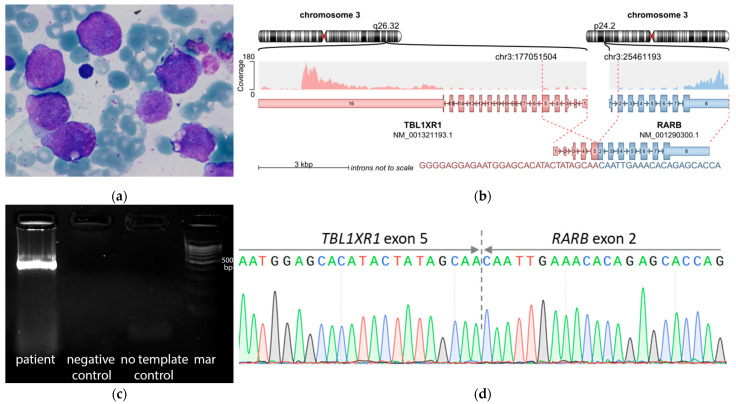
*RARA*-negative AML demonstrating (**a**) blasts with APL-like morphology as depicted by Pappenheim–Kryukov staining in BM smear, (**b**) *TBL1XR1::RARB* fusion transcript in RNAseq data, (**c**) RT-PCR and (**d**) Sanger validation on *TBL1XR1::RARB* fusion transcript (for primers, see Table S1).

*TBL1XR1::RARB*-positive sample was found to have conventional hypergranular M3 morphology (Figure 1a) and exon 5–exon 2 breakpoint junction (Figure 1b–d). Tumor immunophenotype was characterized by a strong expression of conventional myeloid antigens (CD13, CD33, CD15, MPO), although no monocytic (CD14, CD64, CD11c, CD11b, lysozyme), megakaryoblastic (CD61, CD41) and lymphoid (CD7, CD19, iCD79a, CD56, CD2) markers were found. Leukemic cells were HLA-DR-negative, although another known sign of APL, CD117-positivity, was not found. Among precursor markers, CD34 was negative, while strong expression of CD99 was detected.

**Figure 2:**
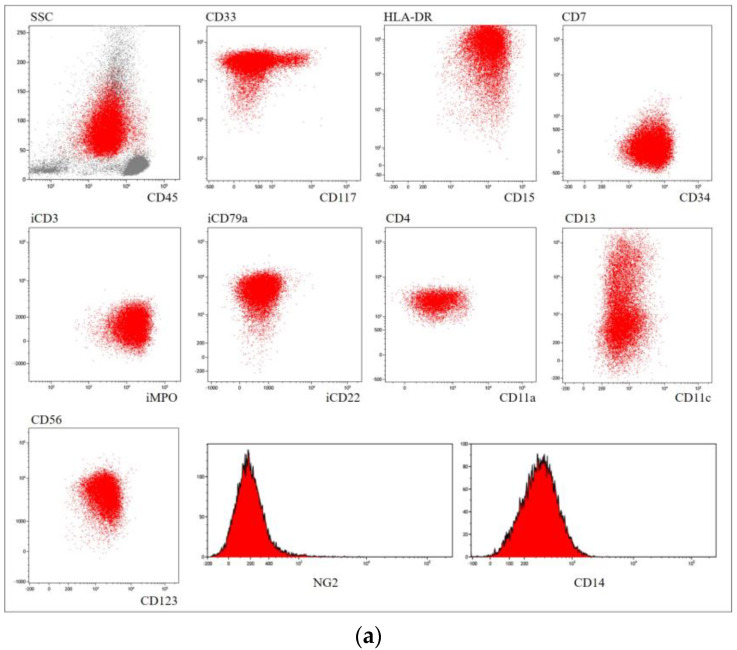

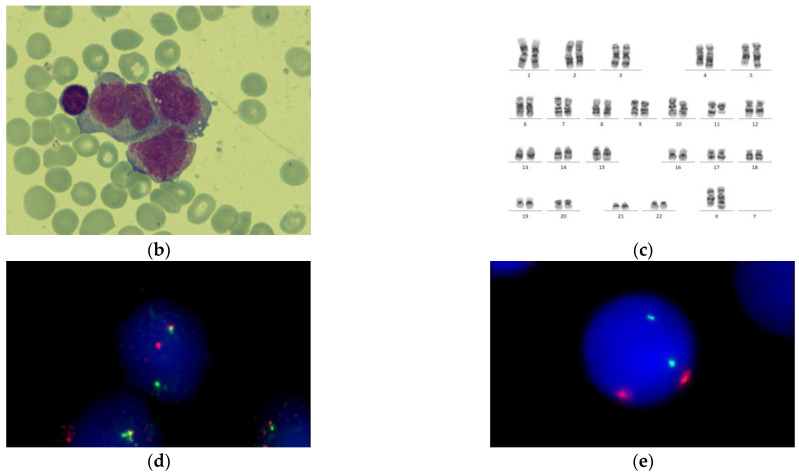

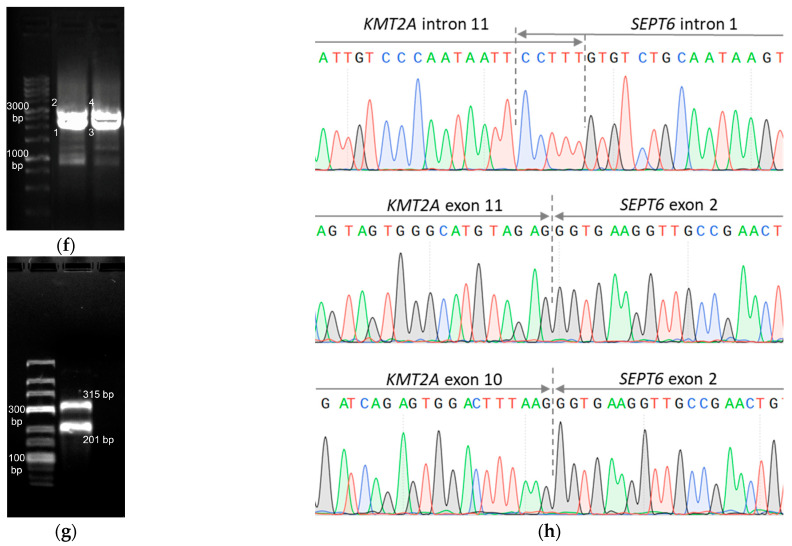
*RARA*-negative *KMT2A*-positive AML patient analysis: (**a**) BM cells immunophenotyping (surface and intracellular (“i”) expression of characteristic markers by flow cytometry; on the dot plots, tumor cells are red while remaining BM cells are gray); (**b**) blasts with APL-like morphology as depicted by Pappenheim–Kryukov staining in BM smear; (**c**) GTG-banded karyotyping showing ins(X;11)(q24;q14q25); (**d**) FISH with Kreatech ON *KMT2A* break-apart probe; (**e**) FISH with Kreatech ON t(15;17) *PML::RARA* dual-color dual-fusion probe; (**f**) *KMT2A* LDI-PCR on BM DNA (2 and 4 are wild-type bands, 1 and 3 are rearranged bands); (**g**) RT-PCR on BM RNA (315 bp band refers to KMT2A::SEPT6 exon 11–exon 6 fusion transcript, 201 bp band refers to KMT2A::SEPT6 exon 10–exon 6 fusion transcript); (**h**) Sanger validation of *KMT2A::SEPT6* fusion gene, KMT2A::SEPT6 exon 11–exon 6 fusion transcript, KMT2A::SEPT6 exon 10–exon 6 fusion transcript (top-down). For primers, see Table S1.

Another *PML::RARA*-negative AML demonstrated numerous promyelocytes with bilobed nuclei and hypogranular vacuolated cytoplasm (Figure 2b). MPO and SB staining were strongly positive in the leukemic cells. The morphologic and cytochemical findings suggested AML of the M3v subtype. Flow cytometry revealed the population of leukemic cells positive for CD45, CD33, CD34, CD15, HLA-DR, CD123, CD4 and MPO; slightly positive for CD11a, CD11c and CD13; negative for CD117, CD14 and NG2, with co-expression of CD56 and cytoplasmic CD79a. This phenotype was not characteristic for APL (Figure 2a). Conventional karyotyping revealed ins(X;11)(q24;q14q25) (Figure 2c). Blast cells contained *KMT2A* gene rearrangement and no t(15;17)(q22;q21) (Figure 2d,e). None of the PML::RARA fusion transcript types were found by RT-PCR. Eight of the most frequent *KMT2A* fusions with partner genes *EPS15*, *AFF1*, *MLLT3*, *MLLT1*, *AFDN*, *MLLT10*, *MLLT6* and *ELL* were also excluded. *KMT2A* intron 11 was fused to the *SEPT6* intron 1 as found by LDI-PCR (GenBank MH973319.1; Figure 2f,g). RNAseq showed the expression of two KMT2A::SEPT6 fusion transcript variants. The Sanger sequence analysis of the products confirmed the identity of the chimeric transcript by showing *KMT2A* exon 11 fused to *SEPT6* exon 2 and an alternatively spliced transcript of *KMT2A* exon 10 fused to *SEPT6* exon 2 (Figure 2g,h). The exact same fusion gene was found in relapse in BM blasts as well as in cells from cerebrospinal fluid.

The remaining five patients did not carry any gene fusions (1.8%; Table S2). All these patients were positive for CD33, CD13 and MPO, while negative for HLA-DR. CD117-positivity was detected in three of them, while one child was CD34-positive as well. Expression of CD123 was found in four patients, CD15 in three, and lysozyme displayed low expression in all three patients who were tested. Only in one case lymphoid antigen (CD2) was found.

Fusion-negative AML with APL-like morphological features did not carry any recurrent chromosomal aberrations. They included two cases of non-recurrent chromosomal rearrangements and three AMLs with normal karyotype. Additional mutations affected *PTPN11*, *NRAS*, *IDH2* and a single case of *NPM1* insertion and *FLT3-ITD* co-occurrence. Nonsynonymous deleterious exonic variants are listed in Table S3. The overall morphological, immunophenotypic and molecular heterogeneity of pediatric AML with atypical promyelocytes accumulation is summarized in Table 1.

**Figure 3:**
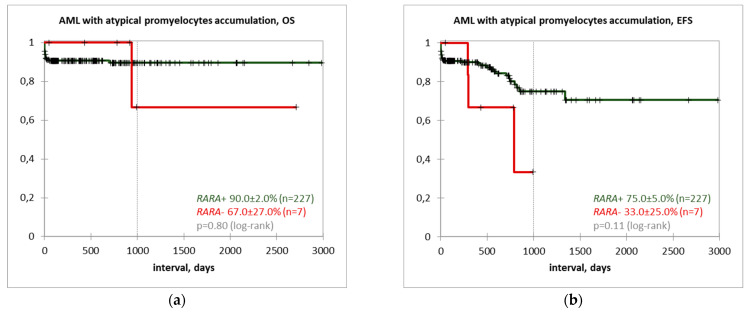
Survival data of pediatric AML with atypical promyelocytes accumulation: (**a**) three-year OS; (**b**) three-year EFS.

Outcome data were available for 86.3% of the studied cohort (234 of 271). The three-year OS rate in patients with *RARA* gene rearrangements placed on ATRA combined with cytarabine and daunorubicin therapy was 90.0 ± 2.0%, while EFS was 75.0 ± 5.0% (*n* = 227). *RARA*-negative patients were treated with either APL approach (same as *RARA*-positive group; *n* = 6) or AML intermediate risk approach (cytarabine, mitoxantrone, etoposide; *n* = 1). *RARA*-negativity was associated with lower survival rates (OS 67.0 ± 27.0%; EFS 33.0 ± 25.0%); however, the difference did not reach statistical significance due to the small size of the *RARA*-negative cohort (*p* = 0.8 and 0.11, respectively). Survival curves are depicted in Figure 3. First-line treatment and outcome data of pediatric AML with atypical promyelocytes accumulation are summarized in Table 1.

## 4. Discussion

Atypical promyelocytes accumulation, as defined by morphology, strongly correlates with the *RARA* gene rearrangements. In our study, 94.1% of patients demonstrated classic APL with t(15;17)(q24;q21)/*PML::RARA*, 1.9% carried cryptic *PML::RARA* and 1.5% had t(5;17)(q35;q21)/*NPM1::RARA* variant (255, 5 and 4 of 271, respectively). *PML::RARA* bcr1:bcr2:bcr3 ratio was reported to be roughly 2:1:2 in both adult and pediatric APL [10,33]. In our cohort, the major subtype was bcr3 accounting for 50.3% of studied cases. The information on transcript subtypes was used for subsequent MRD monitoring by qRT-PCR which is a current standard for this type of AML [34].

NPM1::RARA fusion transcript was found in four patients and exhibited a conservative exon 4–exon 3 breakpoint junction. *NPM1* gene at 5q35 encodes the nucleophosmin protein and moves between the nucleus, nucleolus and cytoplasm, participating in the regulation of p53, the tumor suppressor ARF and many other cellular functions [35,36]. NPM1::RARA acts as a retinoic acid-dependent transcriptional activator similarly to PML::RARA [5]. However, rare cases of *RARA*-negative AML with APL-like features were also described featuring *RARB* or *RARG* [16,37,38,39,40,41,42,43,44,45,46,47], *KMT2A* fusions [33,48,49] and various other genetics [15,33,50,51,52] (see Table S4). In our study, they accounted for 4.4% of the studied cohort and included one case of *RARB* isotype gene rearrangement *TBL1XR1::RARB* (0.4%), one case of *KMT2A*-rearranged AML (0.4%) and five patients without fusions (1.9%).

*TBL1XR1* (transducin β-line 1 X-linked receptor 1) is located at 3q26 and encodes WD-40 protein, which has the LiSH domain [53]. Transcription of this gene affects the interaction of its products with some histones and N-COR and SMRT corepressors, reducing their repressive functions. *TBL1XR1::RARB* transcript discovered in the study had breakpoints in exon 5 and exon 2, respectively, which was previously described in APL [41,43]. The oncogenic protein is similar to *PML::RARA* in its action and reduces the transcriptional activity of the retinoic acid pathway [33].

An unusual case of *KMT2A::SEPT6*-positive AML resembling APL at diagnosis deserves special attention. *KMT2A*, also known as *MLL*, located at 11q23, encodes a lysin-specific histone methyltransferase critical for the *HOX* genes expression. As a result of *KMT2A* rearrangements, upregulated *HOX* expression in hematopoietic stem cells leads to disruption of their differentiation and development of leukemogenesis [54]. Septin genes are rarely but recurrently translocated to *KMT2A*, and *SEPT6* is the most widespread among them [55]. They encode ubiquitously expressed small GTP-binding proteins that are involved in various cellular processes, including cytoskeletal organization, cytokinesis and membrane dynamics [56].

Although most pediatric patients with *KMT2A* rearrangements were diagnosed with acute lymphoblastic leukemia, it was suggested that the *KMT2A::SEPT6* fusion gene tends to induce differentiation of cells into the myeloid lineage [57]. The described patient, being a 3-year-old girl, falls within the characteristic age group. She lacked hepatosplenomegaly, but showed central nervous system (CNS) involvement both initially and in relapse, where it was especially prominent and resulted in left-side face paralysis.

There is a limited number of reports on *KMT2A*-rearranged AML with morphological features resembling APL but without *RARA* rearrangement [33,48,49]. None of them carried *KMT2A::SEPT6* fusion.

Despite the cytomorphological characteristics typical of APL, leukemia cells in the reported case lacked the signature characteristic immunophenotypic signs of malignant promyelocytes observed in APL, i.e., they were positive for HLA-DR, CD34 and brightly positive for CD117. On the other hand, they exhibited typical features of *KMT2A*-rearranged AML, such as very bright expression of CD33 and HLA-DR and expression of CD4 and CD56, which have been noted [58]. NG2 expression, which is considered very specific for aberrations involving *KMT2A* [59,60], was not found on the tumor cells. Thus, being not typically promyelocytic by immunophenotype, the leukemia described did not represent all characteristic *KMT2A*-rearrangement antigen profiles as well.

The leukemic cells of the other six *PML::RARA*-negative AML cases with APL-like morphological features, except the *KMT2A*-positive patient (due to the specific tumor biology), had more or less a typical myeloid immunophenotype, with strong expression of pan-myeloid antigens (CD33, CD13, MPO). Moreover, all of them were HLA-DR-negative as can be expected for APL cases [61]. On the other hand, it is known that HLA-DR-negativity is frequently found in non-APL cases of AML [62]. The expression of CD117, which is also frequently described for APL [63], was found only in part of the cohort, and in several cases progenitor markers (CD99 and CD34) were also expressed. Partial expression of lysozyme is not typical for APL as well. Overall, it can be summarized that *PML::RARA*-negative AML cases with APL-like morphological features displayed relatively heterogeneous antigen expression profiles with a mix of typical and atypical APL immunophenotypic features.

Modern therapy for classical APL with *PML::RARA* is considered to be a combination of ATRA and ATO which both lead to disruption of the fusion protein and restore the activity of the retinoic acid receptor pathway, unblock myeloid differentiation and decrease the proliferative capacity of abnormal cells [2,4,64,65,66]. ATRA, in combination with traditional cytotoxic chemotherapy, has been shown to significantly improve APL outcomes in both adults and children with a 10-year OS of 89% and a 10-year EFS of 76%, as demonstrated by the GIME-MA-AIEOP AIDA 0493 study [67]. We have achieved comparable results (a 3-year OS of 90% and a 3-year EFS of 75%) with this treatment approach.

Variant *X::RARA*-associated APLs are considered to have the same pathogenetic mechanism and were shown to respond to ATRA therapy. However, *NPM1::RARA* cases require a higher dosage of ATRA due to the high affinity for corepressor molecules and are at higher risk of relapse [64,66,68,69]. *PLZF::RARA*, *STAT3::RARA*, *STAT5B::RARA*, or *CPSF6::RARA* were shown to have poor response to ATRA [70]. On the other hand, in some cases of *TBL1XR1::RARA*, remission was achieved after prolonged treatment with ATRA, ATO and chemotherapy [71]. As for the cohort described in the present work, survival data were available for two *NPM1::RARA*-positive patients who received ATRA and chemotherapy, who reached the first CR confirmed by RT-PCR and were alive in CR for 28 and 44 months.

The limited data on ATRA therapy in APL-like AML with retinoic receptors B and G isotypes involvement show their adverse clinical effects. *RARB* and *RARG* demonstrated ATRA resistance and frequent relapses [33,37,41,70]. A complex case was described of a patient with *NPM1::RARG::NPM1* fusion and M3 morphology who had clinical resistance to ATO and ATRA drugs due to the lack of binding sites for these substances [16]. Thus, in the treatment of atypical APL, the effectiveness of the ATRA-based regimen is strictly individual, and HSCT may be accounted as an option. Our reported *TBL1XR1::RARB*-positive patient received APL induction therapy, achieved morphological remission, was set to HSCT but was lost for follow-up 2 months after diagnosis.

ATRA therapy is even more questionable in AML with APL-like morphological features but without any trans-retinoic acid targets. *KMT2A::SEPT6*-positive AMLs meet adverse risk stratification criteria, and intensive induction therapy followed by HCST is a generally recommended treatment option [72]. As for molecular features, the reported case carried *KMT2A::SEPT6* fusion with *KMT2A* intron 11 involvement which was reported to attribute to the lowest survival rate [73]. Our reported *KMT2A::SEPT6*-positive patient started treatment with ATRA combined with cytarabine and daunorubicin. Complete remission was achieved and confirmed by BM morphology and qRT-PCR for KMT2A::SEPT6 fusion transcript. The patient received chemotherapy consolidation consisting of two additional courses of cytarabine and daunorubicin and was placed on maintenance therapy with daily mercaptopurine, weekly methotrexate and ATRA for 2 weeks every 3 months. Due to initial CNS involvement (two cells per mm3 and 64% blasts in cerebrospinal fluid tap) the patient was also placed on CNS therapy consisting of five intrathecal injections of cytarabine, methotrexate and dexamethasone after the onset of the disease. Six months after remission, the patient presented with left-side face paralysis and was hospitalized. Combined BM and CNS relapse was diagnosed confirmed by BM morphology, qRT-PCR for KMT2A::SEPT6 fusion transcript and direct genomic PCR for *KMT2A::SEPT6* fusion gene in cerebrospinal fluid. The patient received high-dose cytarabine and anthracycline without ATO; however, the leukemia proved to be resistant and the patient died because of the progression of the disease.

Cases mimicking various features of acute promyelocytic leukemia that do not carry any fusion genes were also reported [15,33,51]. Arana Rosainz et al. reported a small series of adult AML with immunophenotypic features of APL but without gene fusions, where all patients harbored *NPM1* exon 12 insertion accompanied by *FLT3*-ITD in 60% [15]. A case by Sun et al. was also *NPM1* and *FLT3*-ITD-positive [51]. Oppositely, a larger series by Zhao et al. containing 12 AMLs with morphological features of APL without gene fusions exhibited only one *NPM1* and *FLT3*-ITD-positive case [33]. *RARA*-negative AML with APL-like features described in the literature are summarized in Table S4. Overall, neither cytogenetics nor molecular genetics revealed any characteristic features of this group in our data either; only one patient of five was *NPM1* and *FLT3*-ITD-positive and the rest carried various other mutations.

Regarding therapeutic options, these AML should not be classified as APL, but as intermediate-risk AML according to the ELN recommendations [72]. APL-targeted treatment results in a higher induction failure rate and lower survival rates in APL-like AML compared to *PML::RARA*-positive APL [33]. However, since APL is associated with significant early mortality due to complications of coagulopathy and differentiation syndrome [74], it is highly desirable to start treatment as soon as possible. If any available cytogenetic or molecular method fails to subsequently demonstrate *RARA* rearrangement, Zhao et al. show a possible approach to transition to AML mode (not M3) starting from the second induction course after the first APL induction course. This resulted in an improved survival of patients who switched to an AML regimen, compared to patients treated with an APL regimen, although the difference was not statistically significant [33]. In our cohort, four patients received ATRA combined with cytarabine and daunorubicin (same as *RARA*-positive group) and one patient received cytarabine, mitoxantrone and etoposide (as intermediate risk AML). All patients achieved CR. Remission status was confirmed by flow cytometry, as a unified molecular assessment could not be used in this group. Later, two patients relapsed (9 months after diagnosis each) and were subjected to hematopoietic stem cell transplant. Three patients stayed in the first CR for 14, 25 and 32 months.

The literature data, as well as our own data, suggest that AML mimicking the morphological, cytochemical and immunophenotypic features of APL may arise from different mechanisms other than *PML::RARA* gene formation, and are a heterogeneous AML subgroup. Thus, confirmatory molecular methods are obligatory for chromosomal translocations verification in patients with APL-like features. Our study adds some important traits to characteristics of AML with APL-like features which might help to select appropriate therapy.

## Figures and Tables

**Table 1:** Clinical and molecular-genetical characteristics of pediatric *RARA*-negative patients having AML with atypical promyelocytes accumulation.

N	57	93	94	172	194	235	242
**Age, years**	8	14	3	8	0	1	1
**Sex**	F	M	F	M	M	F	F
WBC *	79	3.4	7.1	2.2	113	55	60
**BM morphology**	M3v	M3v	M3v	M3	M3	M3	M3
**Atypical promyelocytes**	54.5%	78%	94.5%	89%	85.5%	55%	98.5%
**Immunophenotype**	CD33+ CD13+/− CD15+/− CD64+ CD117+ MPO+ CD123+ CD11c+/−	CD33+ CD13+ CD34+ CD2+ CD117+ MPO+ CD123+/−	CD33+ CD34+ CD15+ CD13+/− CD123+ CD56+ MPO+ iCD79a+ HLA−DR+	CD33+ CD13+ CD117+/− MPO+ CD123+ Lysozyme+/−	CD33+/− CD13+ CD15+/− CD99+ MPO+ CD123+/− Lysozyme+/−	CD33+/− CD13+ CD15+/− CD99+ MPO+ Lysozyme+/−	CD33+ CD13+ CD15+ CD99+ MPO+
**Cytogenetics**	NK **	der(8)t(8;8)(q24;q12), der(11)t(8;11)(q12;p15)	ins(X;11) (q24;q14q25)	i(17)(q10), del *TP53*	NK	NK	NK
**FISH for t(15;17)**	negative	negative	negative	negative	negative	negative	negative
**RT-PCR for *PML::RARA***	negative	negative	negative	negative	negative	negative	negative
**Fusion gene**	no fusions	no fusions	*KMT2A::SEPT6*	no fusions	no fusions	no fusions	*TBL1XR1::RARB*
**Detected mutations**	*NPM1* p.W288Cfs*12 *FLT3* p.F594_K602dup	-	-	*NRAS* p.G12R	-	-	-
**First-line therapy**	cytarabine, mitoxantrone, etoposide	ATRA, cytarabine, daunorubicin	ATRA, cytarabine, daunorubicin	ATRA, cytarabine, daunorubicin	ATRA, cytarabine, daunorubicin	ATRA, cytarabine, daunorubicin	ATRA, cytarabine, daunorubicin
**Follow-up time**	88 months	30 months	30 months	25 months	31 months	14 months	2 months
**Outcome**	Alive in 3rd CR ***	Alive in 3rd CR	Reached 1st CR, died of relapse	Alive in 1st CR	Alive in 1st CR	Alive in 1st CR	Reached 1st CR, lost for follow-up

* White blood cell count, /ul, ** Normal karyotype, *** Complete remission

## Data Availability

Data are contained within the article or supplementary material. The sequences of *KMT2A* fusion gene and transcript can be found in the GenBank database (http://www.ncbi.nlm.nih.gov/Genbank, accessed on 4 March 2021) under accession numbers mentioned in the paper. High-throughput sequencing data are publicly unavailable due to ethical restrictions and are only available upon request.

## Supplementary Materials

The following supporting information can be downloaded at https://www.mdpi.com/article/10.3390/genes14030675/s1, Table S1: Primers and probes used in the study; Table S2: Patients’ bone marrow morphology, karyotyping, FISH results and fusion genes; Table S3: Patients’ additional mutations in genes significant for myeloid neoplasm development (Qiagen Human Myeloid Neoplasm, Qiagen, Hilden, Germany); Table S4: *RARA*-negative AML with APL-like features described in the literature.
